# Development of a Novel Pharmaceutical Formula of Nanoparticle Lipid Carriers of Gentamicin/α-Tocopherol and In Vivo Assessment of the Antioxidant Protective Effect of α-Tocopherol in Gentamicin-Induced Nephrotoxicity

**DOI:** 10.3390/antibiotics8040234

**Published:** 2019-11-25

**Authors:** Mahmoud A. Elfaky, Abrar K. Thabit, Alaa Sirwi, Usama A. Fahmy, Raghdah M. Bahabri, Eman A. Al-Awad, Lamis F. Basaeed

**Affiliations:** 1 Natural Products Department, Faculty of Pharmacy, King Abdulaziz University, Jeddah 21589, Saudi Arabia; melfaky@kau.edu.sa (M.A.E.); asirwi@kau.edu.sa (A.S.); 2 Pharmacy Practice Department, Faculty of Pharmacy, King Abdulaziz University, Jeddah 21589, Saudi Arabia; akthabit@kau.edu.sa; 3 Pharmaceutics Department, Faculty of Pharmacy, King Abdulaziz University, Jeddah 21589, Saudi Arabia; 4 Faculty of Pharmacy, King Abdulaziz University, Jeddah 21589, Saudi Arabia; rrr.90952@gmail.com (R.M.B.); eiman.adh.15@gmail.com (E.A.A.-A.); lamisfob@gmail.com (L.F.B.)

**Keywords:** aminoglycoside, gentamicin, α-tocopherol, nephrotoxicity, nanolipid carriers

## Abstract

Gentamicin is a potent antibiotic with a nephrotoxicity drawback which limits its use. D-α-tocopherol polyethylene glycol succinate (α-tocopherol) is widely used as a surfactant and have potent antioxidant properties. This study aimed to assess the protective effect of α-tocopherol on gentamicin-induced nephrotoxicity by loading gentamicin on nanostructured lipid carriers (NLC). In vivo, the product was administered intravenously to three groups of rabbits (control, gentamicin and gentamicin/α-tocopherol NLC) for 10 consecutive days. Blood was collected on days 1, 5 and 10 to assess renal function. A significant difference in all plasma parameters related to kidney function were observed in the gentamicin group compared to the control by day 5 and 10, confirming the nephrotoxicity effect. On the other hand, the same parameter levels of the NLC group were significantly different compared to the gentamicin group, confirming the protective effect on kidney function. Gentamicin also caused significant decreases in plasma levels of glutathione sulfhydryl (GSH) and superoxide dismutase (SOD) activity. However, gentamicin-α-tocopherol NLC significantly elevates both plasma levels of GSH as well as SOD activity. The present work indicates that, loading of gentamicin on NLC by using α-tocopherol, is an innovative strategy to protect against aminoglycoside-induced nephrotoxicity due to its antioxidant activity.

## 1. Introduction

Aminoglycoside is one of the potent bactericidal antibiotics that have been used since 1944 against Gram-negative bacteria and synergistically with β-lactam antibiotics against some Gram-positive bacteria [1,2,3]. Gentamicin is one of the most commonly used aminoglycosides in the clinical setting. Despite the numerous advantages associated with this class of antibiotics, such as the post-antibiotic effect, low cost and low rate of microbial resistance, nephrotoxicity remain one major serious side effect of aminoglycosides contributing to their limited use in the current medical practice with an incidence reaching 10–25% [4,5,6,7].

The mechanism by which aminoglycosides cause nephrotoxicity has been explained by multiple potential mechanisms; however, renal tubular toxicity is the primary mechanism used to describe the nephrotoxic effect of gentamicin [7]. When gentamicin concentration exceeds the threshold in the renal tubules, it disrupts their endosomal structures resulting in the release of cytosol that integrates with gentamicin and act on the mitochondria to ultimately result in necrosis and apoptosis. While hypocalcemia, hypomagnesemia and proteinuria are early signs of nephrotoxicity damage, elevated serum creatinine is considered a sign of severe renal damage [8,9]. Additional nephrotoxic mechanisms include increasing the level of intracellular calcium leading to stimulation of mesangial contraction and decreased glomerular filtration rate, as well as preventing electrolytes and fluid excretion [10,11]. Published studies have shown gentamicin stimulates the production of free radicals, such as relative oxygen species (ROS), which induce renal injury via peroxidation of cell membrane lipids, protein denaturation and DNA damage [3,12,13]. Hence, scavenging ROS ameliorates gentamicin nephrotoxicity by zinc-induced metallothionein synthesis [14].

Antioxidants such as vitamin E (α-tocopherol) have been used as a protective agent against ROS as it has been shown to inhibit peroxidation by hunting lipid peroxyl and hydroxyl radicals [15]. Vitamin E was also shown to mitigate the suppression in enzymatic activity of SOD and GSH in the renal tissues induced by gentamicin [16]. Previous studies have reported that the co-administration of vitamin E and gentamicin significantly prevented nephrotoxicity by the preservation of glomerular filtration rate and renal tissue glutathione [17].

Nanostructured lipid carriers (NLC) are a type of nanoparticle considered as the second generation of intelligent drug carrier systems, and have a solid form in room temperature [18]. They are manufactured by physiological, biodegradable lipid materials that have several features, such as a wide range of usage, compatibility with body fluids and high efficacy to increase the bioavailability of low soluble drugs [19]. NLC received increased attention in the last few years due to their physicochemical stability, biocompatibility, biodegradability and controlled drug release [19,20].

Aminoglycoside-induced nephrotoxicity is not solely an adverse clinical outcome but can also be an economic burden. An old study examined the economic consequences posed by aminoglycoside-induced nephrotoxicity. The authors found that the total cost of the additional care needed by patients who developed renal impairment post treatment with aminoglycosides was $2501 with an average cost of $183 for each patient receiving them [21]. A few published in vivo studies revealed the beneficial effect of combining α-tocopherol with gentamicin on reducing the oxidative stress occurring in gentamicin-induced renal failure. However, all these studies administered the two components separately [17,22,23]. 

From a clinical standpoint, having a single pharmaceutical product containing both entities can help simplifying drug administration, be a safer alternative product of gentamicin, as well as be a cost-effective option given the economic burden that can result from gentamicin-induced nephrotoxicity. Therefore, the objective of this study is to develop a new pharmaceutical formula of nanoparticle lipid carriers combining both α-tocopherol and gentamicin and experiment with it in vivo using a rabbit cyclosporine-induced nephrotoxicity model.

## 2. Results

### 2.1. Characteristics of the Nanostructured Lipid Carriers

**Figure 1:**
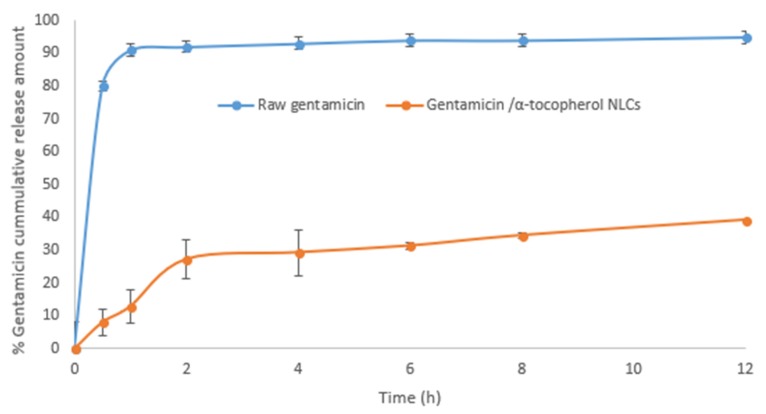
Diffusion profile of raw gentamicin and gentamicin/α-tocopherol nanostructured lipid carriers (NLC).

The prepared NLC particle size was 285.2 ± 14.1 nm with a polydispersity index of 0.51 and a zeta potential of +21.1 ± 3.4 mV. The drug encapsulation efficiency (EE%) of the prepared NLC was 12.2% ± 1.1%. Figure 1 shows the diffusion profile of raw gentamicin and gentamicin/α-tocopherol NLC. 

**Figure 2:**
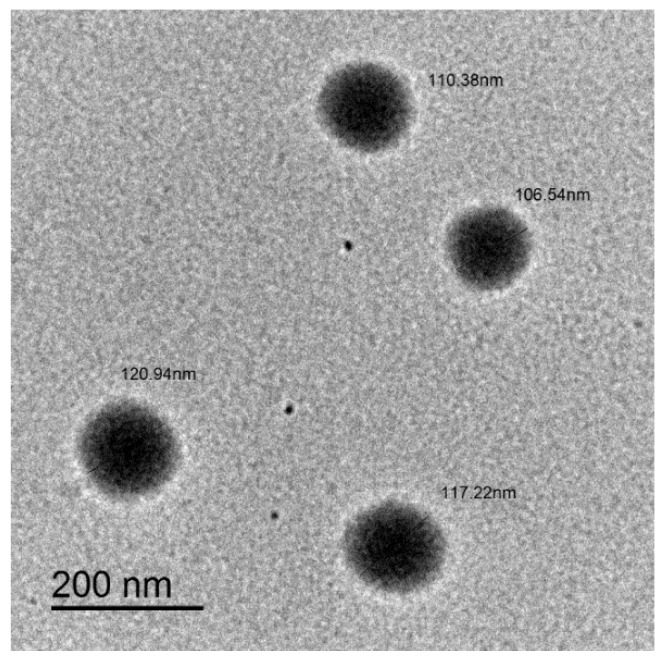
Transmission electron microscopy image of gentamicin/α-tocopherol NLC particles showing their particle size in nanometers.

Raw gentamicin shows a fast dissolution rate of approximately 93% within one hour, which is attributed to the water solubility of gentamicin. On the other hand, gentamicin/α-tocopherol NLC experienced an initial burst effect followed by slow release. Transmission electron microscopy imaging of the NLC revealed spherical particles that have regular edges (Figure 2).

### 2.2. In Vivo Nephrotoxicity Study

**Figure 3:**
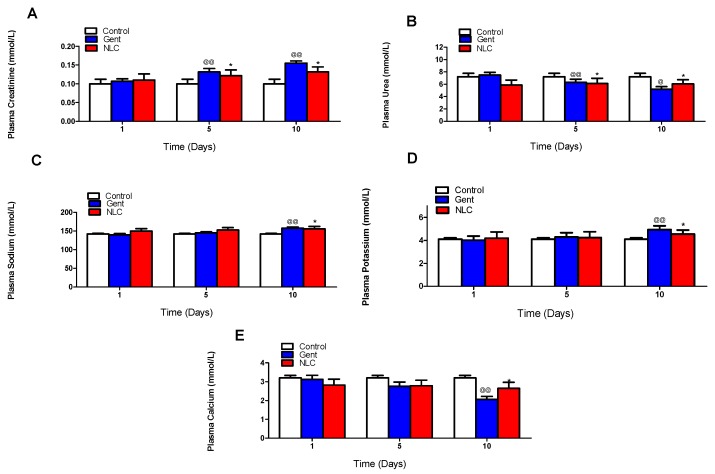
Kidney function parameters measured in rabbit plasma for control, gentamicin and gentamicin-α-tocopherol NLCs. Rabbits (male adult, *n* = 6/group) were IV injected with either normal saline 1 mL every 12 hours (control group), gentamicin 20 mg/kg every 12 hours (Gent group) or the NLC product of gentamicin and α-tocopherol 120 capillary tubes on days 1,5 and 10. The following kidney function parameters were measured from the serum: (**A**) creatinine (**B**) urea (**C**) sodium (**D**) potassium and (**E**) calcium were quantified. Data are presented as mean ± SE (*n* = 6). @ Significant control versus Gent (@ *p* < 0.01 and @@ *p* < 0.001). * Significant Gent versus NLC (*p* < 0.01) determined by Student’s tests.

The in vivo nephrotoxicity study showed significant differences in plasma creatinine, urea, sodium, potassium and calcium between the control and the gentamicin groups, which confirm the nephrotoxicity effect of the drug by long exposure in day 5 and 10 (*p* ≤ 0.01; Figure 3). On the other hand, there were also a significant difference between the gentamicin and the gentamicin/α-tocopherol NLC formula groups in all the plasma parameters by day 10 (*p* ≤ 0.01), as presented in Figure 3. This observation confirms the nephrotoxicity of gentamicin and the protective effect of α-tocopherol against gentamicin-induced nephrotoxicity.

**Figure 4:**
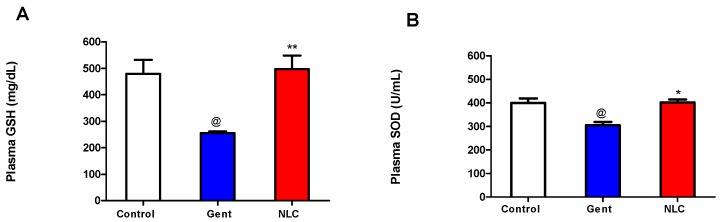
Effect of control, gentamicin (Gent) and gentamicin-α-tocopherol (NLC) on (**A**) glutathione peroxidase (GSH) and (**B**) superoxide dismutase (SOD) content measured in experimental rabbits. Data are presented as mean ± SE (*n* = 4).). @ Significant control versus Gent (*p* < 0.01). * Significant Gent versus NLC (*p < 0.01 and **p < 0.001) determined by Student’s tests.

To determine the mechanism by which the NLC formula is protecting the kidney function we measured GSH and SOD. Figure 4 demonstrates that gentamicin treatment depleted the plasma GSH and SOD, respectively, of the mean control group (*p* < 0.01). However, gentamicin-α-tocopherol NLC significantly prevented such alterations in both parameters (*p* ≤ 0.01)

## 3. Discussion

In this study we proposed a combination product containing both gentamicin and α-tocopherol and tested it in vivo. The product was proposed after in vivo studies, as well as in a human study demonstrating nephrotoxicity protection benefits when vitamin E was administered days prior to gentamicin administration [17,22,23,24]. This benefit is presumed to be attributed to the antioxidant effect provided by vitamin E [15,17]. However, the availability of a single pharmaceutical formulation containing both agents would simplify drug administration and probably save drugs, supplies and labor costs associated with administration of two separate products.

Deep gentamicin entrapped in the NLC is presumed to be affected by factors such as the nature of the lipid carrier and formula composition, the pH of the medium, as well as the particle’s size. So, the biphasic release behavior for gentamicin/α-tocopherol NLC could be attributed to the components of NLC (solid and liquid lipids). The higher melting point (solid) lipid crystallizes first with the drug that forms a free or low concentration liquid lipid core. Most of the liquid lipids are localized at the outer NLC shell that is rich in drugs that releases the drug in a fast (burst) pattern at the initial stage. In order to investigate that the antimicrobial activity of gentamicin in the prepared formula is maintained, an in vitro antimicrobial susceptibility study using broth microdilution was carried out where the minimum inhibitory concentrations (MIC) of the NLCs formula was compared with those of gentamicin alone against 75 Gram-negative bacterial bacilli. Results revealed no differences in susceptibility patterns between the tested components. 

In the present study, as expected, all rabbits injected with gentamicin showed nephrotoxic effects. These were evidenced by the significant difference in the levels of urea, potassium, calcium, creatinine and sodium as nephrotoxicity indicators. The differences were significantly reduced in the gentamicin/α-tocopherol NLC formula group compared to the gentamicin group. Several hypotheses explain the mechanism of gentamicin inducing nephrotoxicity as the role of reactive oxygen species. Our findings showed that gentamicin induced renal oxidative stress by decreasing the GSH and increasing the lipid peroxidation as shown by inhibiting the activity of SOD. An α-tocopherol antioxidant effect could mitigate the effect of gentamicin in inducing nephrotoxicity. Our data agreed with the previous work done on the same aspect. Ramsammy et al. [25] concluded that lipid peroxidation is a proximal event in the injury cascade of gentamicin inducing nephrotoxicity. Abel-naim et al. [23] studied the difference between two groups of rabbits where one was administered gentamicin and the other given gentamicin plus vitamin E. Levels of non-protein sulfhydryl (NPSH) and the activity of antioxidant SOD were measured. The authors reported a significant increase in the NPSH level and in the activity of SOD in the gentamicin–vitamin E group. Over the last decades, the use of aminoglycosides has dramatically decreased as newer and safer alternatives have been developed. Nonetheless, due to the emergence of bacterial resistance to antibiotics, aminoglycosides have been reintroduced again in the clinical setting. Besides therapeutic drug monitoring, administering aminoglycosides with α-tocopherol is an innovative strategy to mitigate their nephrotoxicity without compromising the antibacterial effect of gentamicin. The newly developed NLC formula containing gentamicin combined with α-tocopherol can be the next generation of non-nephrotoxic aminoglycosides.

## 4. Materials and Methods 

### 4.1. Materials

Gentamicin sulphate was a gift from Spimaco Aldawaeria (Al-Qassim, Saudi Arabia), and Compritol® (888 ATO) was a gift from Gattefossé (Saint-Priest, France). D-α-tocopherol polyethylene glycol 1000 succinate and almond oil was purchased from (Sigma Aldich, USA) and hydrogenated phosphatidylcholine from soybean was purchased from LIPOID BH, FRISTRASSE, LUDWIGSHAFEN, Germany

### 4.2. Preparation of Nanostructured Lipid Carriers

The lipids mixture was prepared by the hot emulsification–ultrasonication method using the following ingredients: almond oil (0.3 g), lipid Compritol (0.3 g), phospholipid (1.25 g) and gentamicin (0.64 g). All components were placed in a beaker with 25 mL chloroform and stirred with heating up to 80 °C. Afterwards, the organic solvent was removed gradually through evaporation using rotavapor (BUCHI labortechnik AG, Switzerland). α-Tocopherol (0.625 g) was mixed in 30 mL distilled water and heated up to 80 °C with continuous stirring until completely dissolved. The dissolved α-tocopherol solution was then added to the aforementioned lipid formulation while both beakers were still warm. The final suspension product was then warmed in a water bath at 80 °C for 3 min followed by sonication in water for 3 min (QS3 ultrasonic cleaner, UK), and then further ultrasonicated at 35% amplitude for 3 min (Sonics VC750, Newtown, CT, USA) to obtain an oil-in-water emulsion. Finally, the final volume was adjusted to 50 mL using distilled water. All produced NLC were characterized for their size. The emulsion was left to cool at room temperature.

### 4.3. Size and Zeta Potential of the NLC

The dynamic light scattering technique was utilized to measure the particle size and zeta potential of the prepared NLC using the Malvern ZetaSizer (UK). A sample of 2 mL from each nano formulation was taken, loaded into the sampler cuvette, and measured. Measurements were done in triplicates for each formulation and the size/zeta potential averages were recorded.

### 4.4. Drug Content

An indirect method was used to determine the gentamicin drug content. NLC were centrifuged at 20,000 rpm and the supernatant was withdrawn and injected directly to the high-performance liquid chromatography (HPLC) (Agilent Technologies 1100, Germany), using the following equation:

% Drug content = (Initial amount of drug added − Free drug of initial amount of drug added)/(Initial amount of drug added) × 100.

### 4.5. Drug Release Profile

In vitro release studies are one of the most important studies conducted for all sustained drug release delivery systems. Determination of drug release rates from different film formulae was carried out using a USP dissolution test apparatus (Pharmatest, Germany) of a specifically designed glass cylinder. A cellophane dialysis membrane was previously soaked for 12 h in a phosphate buffer (pH = 7). The membrane was then stretched around one end of the tube, making the effective surface area of the membrane equaling 3.14 cm^2^ in diameter. The whole tube (donor compartment) was hung on a rolling stack into a glass beaker (receptor compartment) with the membrane just touching the receptor medium. One square centimeter weighed films of each tested formulation was introduced into the donor tube. The contents of the receptor compartment (500 mL phosphate buffer solution at pH = 7) were thermostatically adjusted to 37 ± 0.5 °C and stirred at 50 rpm adapted with modifications. Aliquots of 1.5 mL samples were withdrawn from the receptor medium at seven time point intervals and replaced with equal volumes of fresh buffer. The drug content was determined and analyzed using HPLC (Agilent Technologies, Germany). The results obtained were the average of three measurements. The condition of the experiment was selected to achieve the physiological sink conditions. The percentage of the released drug was plotted against time.

### 4.6. In Vivo Nephrotoxicity Study

A rabbit cyclosporine-induced nephrotoxicity model was used for the experiment. Three groups of male adult rabbits with a mean weight of 2.5 ± 0.8 kg were used. Each group consisted of six rabbits. Groups received intravenous injections of either normal saline 1 mL every 12 h (control group), gentamicin 20 mg/kg every 12 h (gentamicin group) or the NLC product of gentamicin and α-tocopherol 120 mg/kg every 12 h (NLC group). Animals were obtained from the animal house of the Faculty of Pharmacy, King Abdulaziz University, Jeddah, Saudi Arabia. The in vivo study protocol was approved by the Animal Ethics Committee of the Faculty of Pharmacy, King Abdulaziz University, Jeddah, Saudi Arabia, in adherence with the Declaration of Helsinki, the Guiding Principle in Care and Use of Animals (DHEW production NIH 80-23) and the Standards of Laboratory Animal Care (NIH distribution #85-23, reconsidered in 1985). 

Animals were adapted for at least 2 weeks in naturally controlled enclosures (20 °C ± 1 °C and a 12/12-hour dark/light cycle). Libitum was added to standard food and water. Blood samples were withdrawn from the medial canthus of the eye by means of capillary tubes on days 1, 5 and 10. The following kidney function parameters were measured from the serum: creatinine, urea, sodium, potassium and calcium using a previously described method [26].

### 4.7. Measurement of Lipid Peroxide

Glutathione sulfhydryl (GSH) was measured using Biodiagnostic kits (Egypt). The level of GSH was determined according to the previously described method [27]. This assay was based on the reduction of bis-(3-carboxy-4-nitrophenyl) disulfide reagent by the thiol group to form 2-nitro-5-mercaptobenzoic acid. The absorbance of the yellow color formed was measured spectrophotometrically at 412 nm. 

### 4.8. Measurement of Enzyme Activity of Superoxide Dismutase (SOD)

The activity was determined using Biodiagnostic kits (Egypt). SOD activity was determined according to the previously described method [28]. This assay depended on the ability of the SOD to inhibit the phenazine methosulphate-mediated reduction of nitro-blue tetrazolium dye.

Data were presented as mean ± standard error and were statistically analyzed using one-way ANOVA (SPSS, Inc., Chicago, IL, USA) to measure the differences between the parameters. The confidence level using GraphPad Prism 6 (GraphPad Software, San Diego, CA, USA) was set at * *p* < 0.01 and ** *p* < 0.001.

## 5. Conclusions

Gentamicin NLC formulated by α-tocopherol are a promising formula that exhibited reduced gentamicin nephrotoxicity. This was due to the kidney protective effect of α-tocopherol which elevates plasma levels of GSH and SOD. The NLC group improved kidney function parameters significantly in comparison with raw gentamicin. Further clinical evaluation of this novel formula is necessary to confirm these results and to provide further data.
